# A retrospective analysis of oral and maxillofacial pathology in a pediatric population from Rio de Janeiro–Brazil over a 75-year period

**DOI:** 10.4317/medoral.22428

**Published:** 2018-09-28

**Authors:** Maria-Luiza Prosdócimo, Michelle Agostini, Mário-José Romañach, Bruno-Augusto-Benevenuto de Andrade

**Affiliations:** 1 DDS, MSc. Department of Oral Diagnosis and Pathology, School of Dentistry, Federal University of Rio de Janeiro (UFRJ), Rio de Janeiro, Brazil; 2 DDS, PhD. Department of Oral Diagnosis and Pathology, School of Dentistry, Federal University of Rio de Janeiro (UFRJ), Rio de Janeiro, Brazil

## Abstract

**Background:**

The aim of this study was to analyze the distribution of oral and maxillofacial lesions affecting children and adolescents patients from a single oral pathology laboratory from Rio de Janeiro, Brazil.

**Material and Methods:**

Oral and maxillofacial lesions biopsied in patients younger than 19-years were retrieved from the oral pathology files of the Department of Oral Diagnosis and Pathology, School of Dentistry, Federal University of Rio de Janeiro over a 75-year period (1942-2017). The clinical data and the diagnoses of each case were included in a Microsoft Excel® database, being classified into 13 categories according to the etiology. A descriptive analysis of the variables age, gender and final diagnosis was made.

**Results:**

From 19.095 lesions diagnosed in this period, 2408 (12.61%) were from patients aged 0 to19 years, with a higher incidence in females in the second decade. Salivary gland pathology was the most common group of lesions (24.30%), followed by reactive lesions (16.82%) and odontogenic cysts (14.66%). Mucocele was the most common lesion (21.72%), followed by dentigerous cyst (6.48%) and fibrous hyperplasia (6.44%). Malignant lesions were observed in 1.12% of all cases with Burkitt lymphoma as the most frequent.

**Conclusions:**

Our results were similar to previous studies and knowledge of these data may contribute to the understanding of oral lesions that most commonly affects children.

** Key words:**Pediatrics, children, pathology, oral lesions, oral cavity.

## Introduction

The knowledge of oral and maxillofacial lesions through epidemiologic studies represents an important role in public health, revealing the accuracy of prevalence, incidence and evolution of several diseases that affect the oral cavity as well as the percentage distribution within the characteristics of certain regional and global populations (1,2).

According to WHO criteria of 1986 (3), childhood and adolescence are the period comprising from 0 to 9 years and 10 to 19 years, respectively. Children and adolescents in particular present a large variety and prevalence of oral pathology conditions, with clinical features that are often different from those of adults (1). The occurrence of oral and maxillofacial pathology in children and adolescents is estimated at approximately 7–17% of all assessed specimens, depending on the patient age range of each study (1,2,4-10).

There are few large sample studies of oral lesions in children with more than 2000 cases published in the English-language literature to date, with only two of them being conducted in Brazil (4,10). The objective of this study is to analyze the distribution of oral and maxillofacial lesions in a pediatric population from Rio de Janeiro – Brazil.

## Material and Methods

A retrospective analysis was conducted in the Oral Pathology Laboratory at Department of Oral Diagnosis and Pathology, School of Dentistry of the Federal University of Rio de Janeiro (Rio de Janeiro, Brazil) including patients younger than 19 years-old biopsied over a 75-year period (from January 1942 to December 2017). The diagnoses, age and gender of each patient were retrieved from the laboratory’s files and tabulated in Microsoft Excel® for descriptive analysis. Additionally, the diagnoses were compiled into 13 diagnostic categories adapted from John and Franklin (1) and Ha *et al.* (2) as follows: dental pathology, reactive lesions, benign neoplasms, infectious diseases, odontogenic cysts, non-odontogenic cysts, odontogenic tumors/hamartomas, bone pathology, salivary gland pathology, normal tissue, malignant tumors, autoimmune and immunological disease and miscellaneous pathology. The data were digitized, processed and classified, using the software program SPSS 15.0 for Windows – Statistical Package for Social Science and were organized into tables afterwards. A descriptive analysis of the variables age, gender and final diagnosis are described and discussed in the present study.

## Results

From 19.095 lesions diagnosed in the 75-year period, 2408 (12.61%) affected patients 19 years-old or younger. There were 1300 girls and 1108 boys (F:M proportion of 1,17:1). Most patients was adolescents in the second decade of life (1748 patients, 72.59%) while 660 patients (27.41%) were children in the first decade of life. Mucocele/ranula was the most common oral lesion (523 cases, 21.72%), followed by dentigerous cyst (156 cases, 6.48%), fibrous hyperplasia (155 cases, 6.44%), and pyogenic granuloma (118 cases, 4.90%) ( Table 1).

**Table 1:**
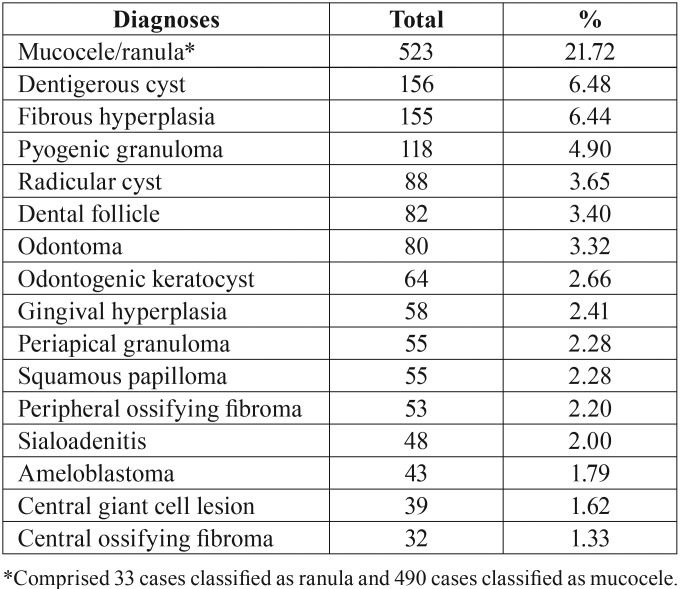
Most frequent oral lesions in pediatric patients from Rio de Janeiro-Brazil.

Salivary gland pathology (585 cases, 24.30%) was the most common group of oral pediatric lesions followed by reactive lesions (405 cases, 16.82%), odontogenic cysts (353 cases, 14.66%), miscellaneous pathology (245 cases, 10.18%), dental pathology (224 cases, 9.30%), odontogenic tumors/hamartoma (154 cases, 6.40%), bone pathology (144 cases, 5.98%), infectious diseases (99 cases, 4.11%), normal tissue (87 cases, 3.61%), benign neoplasms (53 cases, 2.20%), malignant tumors (27 cases, 1.12%), non-odontogenic cysts (16 cases, 0.66%), and autoimmune and immunological disease (16 cases, 0.66%) ( Table 2,  Table 2 continue,  Table 2 continue-1).

**Table 2:**
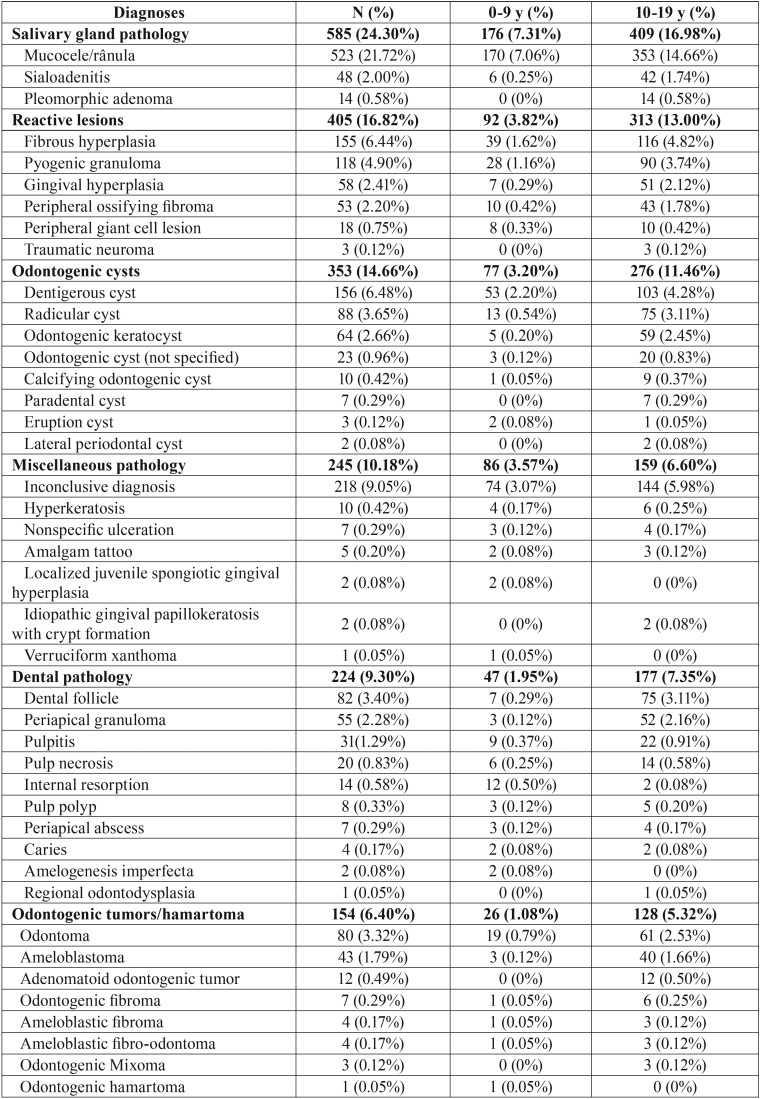
Classification of oral lesions in pediatric patients from Rio de Janeiro-Brazil.

**Table 2 continue:**
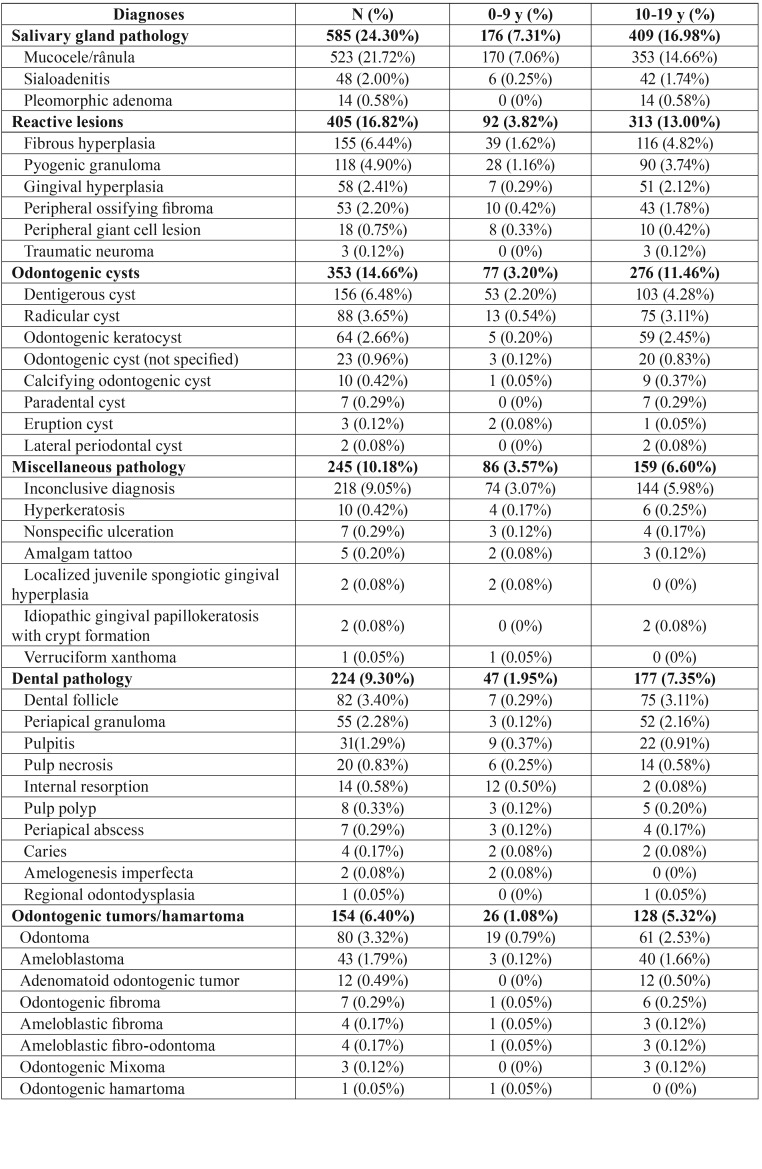
Classification of oral lesions in pediatric patients from Rio de Janeiro-Brazil.

**Table 2 continue-1:**
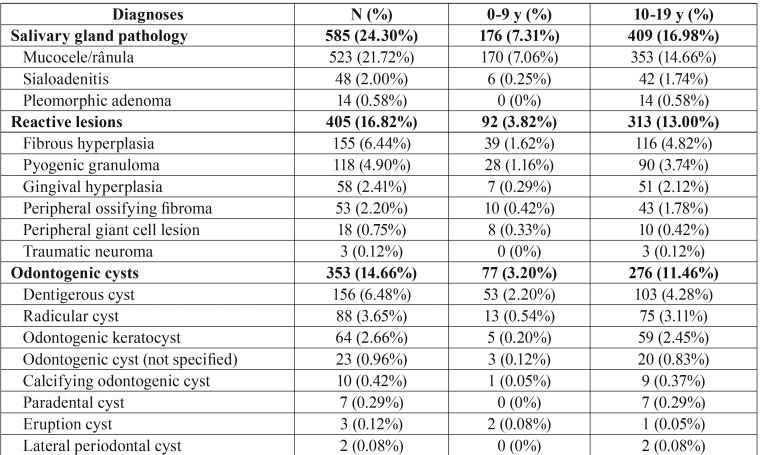
Classification of oral lesions in pediatric patients from Rio de Janeiro-Brazil.

## Discussion

This study provides for the first time a comprehensive analysis of oral and maxillofacial pathology encountered in a children and adolescents population from Rio de Janeiro, Brazil. The prevalence of oral lesions in children and adolescents presents considerable variation according to studies conducted in different geographic regions, with prevalence of all oral lesions ranging between 7 and 17% (1,2,8,9,10). This disparity among the different studies may be due the methodologies used in the literature that are not uniform, including different periods of time, age ranges and diseases categorization, which may become difficult the appropriate analysis of data. In the present study it was observed that 12.61% of the oral and maxillofacial lesions occurred in children and adolescents, a percentage within the range of variation found by other authors (2,7,9,10).

In our cases we found a similar distribution of lesions between both genders, with a slight preponderance for females that accounted for 53.98% of the cases as described by other authors (4,11), although some series observed no predilection for gender (5). The female predominance may be consequence of the parents awareness with their children, since a higher frequency of females is found in adult populations, possibly representing the higher concern demonstrated by females than males (8,9,10). As previously described, we also observed that adolescents present a higher frequency of lesions compared to children, what may be consequence of clinicians behavior, because in the management of pediatric patients, there is a general preference to avoid invasive treatments, which may result in suspected benign conditions being monitored and biopsied later in life when the patient be more compliant; so, the patient age may be a reflection of the age of treatment rather than the time of initial presentation or diagnosis (2,7-10).

Dental pathology represented the most common group of lesions in oral pathology laboratories from Europe, Australia and Thailand while salivary gland pathology was the most common in our study (1,2,11). We believe that this finding more likely represents feature of our laboratory as dental hard tissue abnormalities are usually not frequently submitted to microscopic exam as also observed in other Brazilian studies, suggesting that differences in the frequency of the main oral pediatric lesions might be influenced by characteristics of each diagnostic service (4-10). It is also important to note that the origin of the data retrieved may also influence the results obtained, since surveys performed in oral pathology laboratories of universities are more likely to provide lower prevalence of tumors, when compared with those conducted in hospitals, which show greater prevalence of both benign and malignant tumors (12,13).

The most frequent group of oral lesions observed in our study is in accordance with that observed in other Brazilian studies, with mucocele/ranula, dentigerous cyst and reactive lesions being the most common (4-10). As shown in  Table 3, we tried to identify differences in the frequency of lesions by comparing our results with those previously published in large series of oral lesions in pediatric patients (1,2,4,9,10). There seems to be no study performed in Rio de Janeiro, Brazilian Central and Northern regions, but some studies have described the prevalence of oral and maxillofacial lesions in children and adolescents from Northeast, South and Southeast regions of Brazil (4-10). In these studies children and adolescents accounted from 6.6% to 13.1% of the cases, with a female predominance, and variation on age range from 0-14, 0-16, 0-18 and 0-19 years-old, investigating lesions from 5 to over 20 years period (3-9). Although these variants, only small differences were found in the distribution of the most frequent lesions. As in our study, mucous extravasation cyst predominated in all studies and dental follicle and dentigerous cyst were also common (1,2,4-10).

**Table 3:**
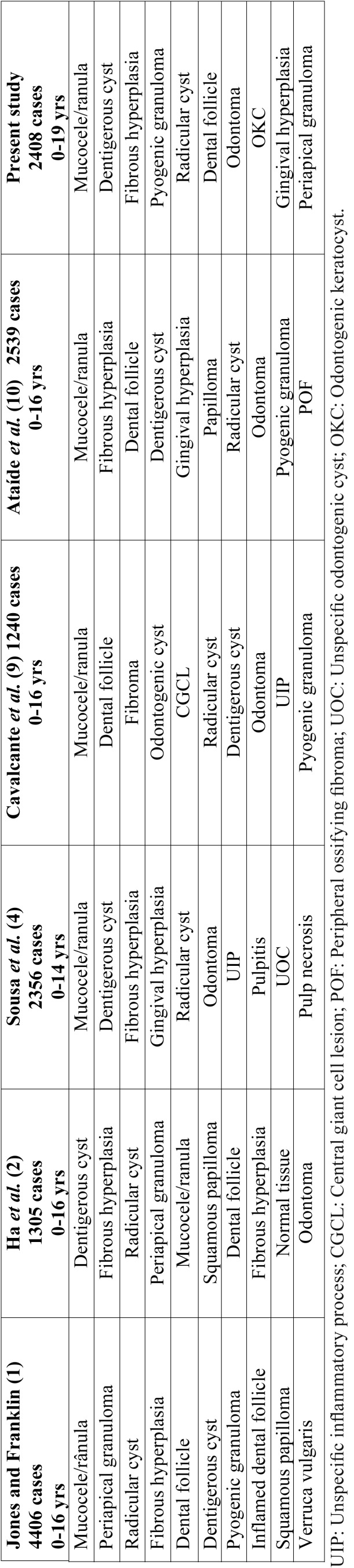
Comparative analysis of large series of oral lesions in pediatric patients including the present study.

Although the majority of lesions reported in children and adolescents are benign and require minimal intervention, it should be recognized that oral malignant tumors do occur in children (1,2), as also observed in our study where 27 cases (1.12% of all cases) were malignancies. In general, malignant tumors in children and adolescents comprise less than 1% of all diagnoses (1). Rhabdomyosarcoma is the most common soft tissue sarcoma of children, adolescents and young adults representing about 3–4% of all cancers affecting children and 35% of cases affecting the head and neck region, like Langerhans cell histiocytosis which also has predilection for pediatric population (14). Our study showed the highest number of malignant tumors cases in children and adolescent among all Brazilian studies (4-10). The most common malignant tumor was Burkitt lymphoma, followed by rhabdomyosarcoma together with Langerhans cell histiocytosis, osteosarcoma and diffuse large B-cell lymphoma. The high prevalence of Burkitt lymphoma is a finding also observed in African studies, suggesting genetic similarities between Brazilians from Rio de Janeiro and African population (15).

In summary, this is the second largest series of oral lesions in pediatric patients from Brazil and the first study evaluating a pediatric population from Rio de Janeiro-Brazil. Mucocele/ranula, dentigerous cyst, fibrous hyperplasia, and pyogenic granuloma were the most common oral lesions. Additionally, we have presented the highest number of malignant tumors in children and adolescents among all Brazilian studies, being Burkitt lymphoma the most frequent. Since retrospective studies of large series of oral lesions in children and adolescents are scarce in the literature, these data may contribute to the understanding of oral pathologists, dental pediatricians, and different medical specialists regarding oral diseases that affect different populations of children and adolescents.
